# Surface-localized thermoresponsive molecularly imprinted polymer for gentamicin recognition and aqueous regeneration

**DOI:** 10.1039/d6ra07976h

**Published:** 2026-10-22

**Authors:** Dominika Rapacz-Kinas, Mikołaj Mroczek, Joanna Wolska, Katarzyna Smolińska-Kempisty

**Affiliations:** a Wroclaw University of Science and Technology, Department of Process Engineering and Technology of Polymer and Carbon Materials Wybrzeże Wyspiańskiego 27 50-370 Wrocław Poland dominika.rapacz-kinas@pwr.edu.pl katarzyna.smolinska@pwr.edu.pl

## Abstract

Gentamicin is a widely used aminoglycoside antibiotic whose selective extraction from aqueous matrices remains challenging because of its high polarity and the presence of structurally diverse coexisting compounds. Molecularly imprinted polymers (MIPs) offer selective recognition of target molecules; however, conventional bulk-imprinted materials may suffer from limited accessibility of recognition sites and relatively long mass-transfer pathways. In this study, a thermoresponsive core–shell MIP was developed for the selective extraction and temperature-controlled regeneration of gentamicin. A PVC-based core was functionalized with a surface-associated molecularly imprinted polymer phase containing poly(*N*-isopropylacrylamide), providing temperature-responsive behavior that enables aqueous regeneration of the sorbent. The performance of the core–shell MIP was compared with that of the corresponding non-imprinted material and evaluated in static sorption and solid-phase extraction experiments. The MIP exhibited a higher gentamicin sorption capacity than the non-imprinted material, reaching 3.90 mg g^−1^ compared with 2.80 mg g^−1^, corresponding to an imprinting factor of 1.4. Temperature-dependent desorption enabled regeneration of the sorbent using cold water, with regeneration efficiencies of 94–100% during the initial cycles. The material also demonstrated preferential recognition of gentamicin in the presence of other antibiotics. The surface-localized architecture distinguishes the present material from the previously reported bulk-imprinted thermoresponsive gentamicin sorbent. The developed approach demonstrates the potential of combining surface molecular imprinting with thermoresponsive polymer functionality for selective solid-phase extraction and aqueous regeneration of gentamicin.

## Abstract 2

Gentamicin is a widely used aminoglycoside antibiotic whose selective extraction from aqueous matrices remains challenging because of its high polarity and the presence of structurally diverse coexisting compounds.

## Graphical abstract

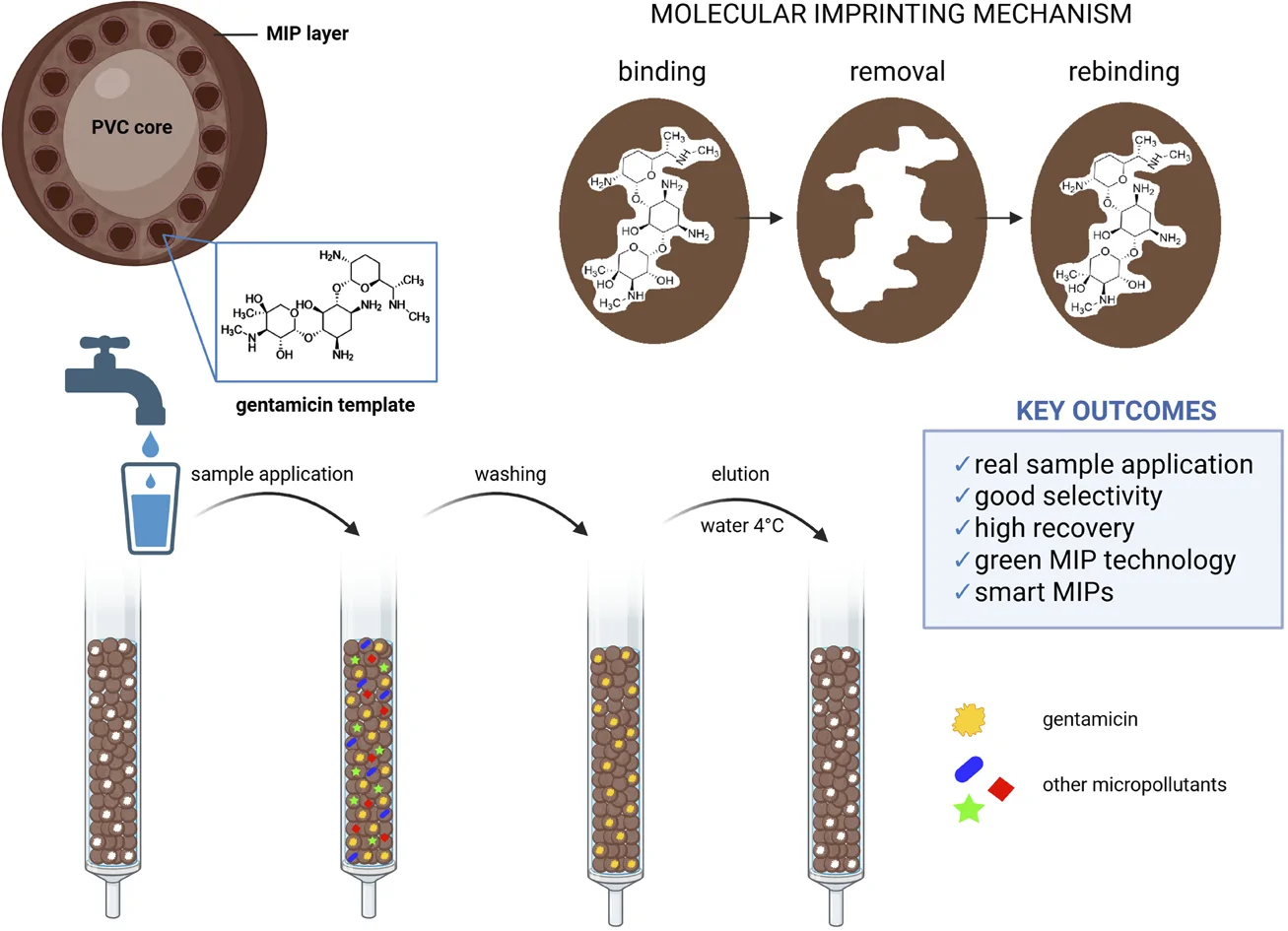

## 1. Introduction

Smart polymers are a type of polymer that undergoes reversible physical, biological, or chemical changes in response to environmental changes. We can distinguish polymers that respond to a single stimulus or those that are programmed to respond to multiple stimuli. Factors that can alter polymer structure include temperature, pH, electric and magnetic fields, light intensity, and biological factors such as enzymes.^1^ Polymers respond to stimuli in several ways, including changes in shape, solubility, wettability, color, conductivity, or permeability.^2^ Exposure to these factors triggers macroscopic reactions in the polymer structure, such as swelling or sol–gel transition.^1^ The degree of response to a given stimulus is regulated by its intensity, resulting in changes in the polymer structure resulting from the formation or degradation of hydrogen bonds, hydrophobic effects, osmotic pressure, and electrostatic interactions.^2^ The specificity of this type of polymers allows for their wide use in various fields of industry and beyond.^2^

In the case of temperature-responsive polymers, we can distinguish two main types: those with a low critical solution temperature (LCST) and those with an upper critical solution temperature (UCST). In the case of LCST, we are talking about the temperature below which the system transitions from a two-phase region to a single-phase region. The situation is opposite for UCST-type polymers.^3–6^

The most popular example of a LCST polymer is poly(*N*-isopropylacrylamide) (PNIPAM). This amphiphilic polymer contains hydrophilic (amine groups) and hydrophobic (isopropyl groups) groups in its monomeric unit. Upon exceeding a temperature of 32 °C, its structure changes from soluble to insoluble in aqueous solution.^7–9^ This effect was exploited to create molecularly imprinted polymers (MIP) capable of multiple reuse.

Molecularly imprinted polymers are synthetic materials designed with specific binding sites that can selectively recognize and bind target molecules. They typically consist of a cross-linked polymer network with cavities that have a high degree of structural specificity.^10–13^ Molecularly imprinted polymers enriched with a core–shell structure represent an advanced form of MIP (CS-MIP). These materials consist of a core that acts as a carrier and imparts specific physical properties to the material. The shell is a thin layer of molecularly imprinted polymer surrounding the core. This layer is responsible for the selective binding of analyte molecules.^14–17^ The use of core–shell architecture allows for increased analyte binding rates due to a short diffusion path. This type of material is therefore used in applications such as liquid chromatography (HPLC) as highly selectivity stationary phases,^18,19^ drug delivery systems (DDS),^20–22^ biosensors and biomolecule detection systems,^23–26^ and environmental and pharmaceutical analysis.^27–29^

Our previous work^30^ demonstrated that thermoresponsive bulk-imprinted polymers can be used for the selective extraction and temperature-induced release of gentamicin in SPE columns. However, in these materials, the molecularly imprinted recognition sites are distributed throughout the bulk polymer matrix, which may limit their accessibility and increase the diffusion path for the target molecule. In that study, the development of a core–shell architecture was identified as a promising direction for further improvement of self-regenerating MIPs.

In the present study, we address this limitation by developing a core–shell molecularly imprinted architecture in which the gentamicin-selective polymer layer is formed at the surface of a PVC core. Unlike the previously reported bulk-imprinted material, the present design localizes the recognition sites within a polymer shell, providing direct access to the imprinted regions and reducing the diffusion distance between the aqueous phase and the recognition sites. The shell additionally incorporates poly(*N*-isopropylacrylamide), allowing the binding environment to be modulated by temperature and enabling regeneration with cold water.

The objective of this work was therefore to determine whether transferring the thermoresponsive gentamicin-imprinted system from a bulk polymer to a core–shell architecture can provide a practically useful sorbent for selective solid-phase extraction, while retaining temperature-controlled aqueous regeneration.

In the final stage of the development, the core–shells were placed in solid-state extraction columns. A sample prepared using this technique transforms the original matrix into a simpler environment, reducing the need for an analytical system. This allows the sample to be better prepared for further chromatographic analysis, often facilitating and increasing the accuracy of the final analysis, both qualitative and quantitative.^31–33^ During the process, a sample is passed through an SPE column, and then the analyte is eluted using organic solvents or, for example, inorganic acid solutions. However, SPE strives for an optimal procedure that ensures the highest efficiency of analyte isolation and purification while minimizing losses, matrix interferences, and solvent consumption.^31^ For this reason, in this publication, smart polymers were used instead of solvent elution. This enabled analyte recovery in a more ecological and environmentally friendly manner. The novelty of the present work lies in transferring the previously developed thermoresponsive gentamicin-imprinted system from a bulk polymer architecture to a surface-localized recognition phase on a PVC support, while retaining PNIPAM-mediated temperature-responsive regeneration in water.

## 2. Materials and methods

### 2.1. Chemicals

Gentamicin sulphate salt (GENT) was purchased from Sigma-Aldrich, amoxicillin sodium salt (AMO) was obtained from MedChemExpress, and ofloxacin (OFX) was used in the form of Floxal eye drops from BAUSCH + LOMB. *N*-isopropylacrylamide (NIPAM), *N*,*N*′-methylenebis (acrylamide) (MBAA), *N*,*N*,*N*′,*N*′-tetramethylethylenediamine (TEMED), ammonium persulfate (APS), isooctane, and potassium hydroxide (KOH) were obtained from Sigma-Aldrich. Acrylamide (AA) was supplied by Thermo Scientific Chemicals. Acrylic acid (AAc) was purchased from Acros Organics. Ethyl alcohol (EtOH) and isopropyl alcohol were obtained from POCH S.A. Poly(vinyl chloride) (Ongrovil® S-5167), which has a medium molecular weight, and with a *K* value of 66–68 (an indicator of molecular weight), was acquired from BorsodChem. The HPLC-grade acetonitrile was sourced from CHEMSOLUTE. Ultrapure water was obtained using a Milli-Q System (Merck Millipore). All of the reagents were of analytical-reagent grade or higher and were used without further purification.

### 2.2. Dehydrochlorination of PVC grains

The dehydrochlorination of PVC was carried out according to the procedure reported by Wolska *et al.*^34^ Briefly, 67.5 g of PVC were dispersed in 500 mL of 10% KOH solution prepared in an isopropanol–water mixture (4 : 1 w/w). This reaction mixture was heated under the reflux at the boiling point for 24 h. During the process, the color of the mixture gradually changed from colorless to dark brown. Subsequently, the degraded PVC was filtered, thoroughly washed with excess distilled water, and finally rinsed with ethanol. The obtained material was then subjected to ethanol extraction for 24 h. After extraction, the dehydrochlorinated PVC (DHPVC) was dried at 60 °C in a dryer until constant weight was achieved.

### 2.3. Preparation of core–shell imprinted and non-imprinted polymers

Core–shell-type molecularly imprinted particles (CS-MIP) were prepared using a surface-imprinting approach. Initially, DHPVC was obtained (the synthesis method was outlined in 2.2.) and subsequently placed in the reaction vessel. The polymerization mixture was then prepared in the following steps. NIPAM (0.04 g), AA (0.033 g), AAc (10 µL), MBAA (0.036 g) and gentamicin (0.012 g) were dissolved in water (9 mL) and added to DHPVC grains (1 g). Then, the initiator system comprising APS (0.022 g) and TEMED (10 µL) in 1 mL of water was introduced, and the mixture was purged with nitrogen for 5 minutes. In the next step, the reaction vessel was placed on a shaker and the process was carried out for 24 hours. After this time, the core–shell particles were filtered through a Buchner funnel and extracted using a Soxhlet apparatus for 24 hours in order to remove the gentamicin molecules. The synthesis of core–shell non-imprinted polymers was conducted in the same way, with the exclusion of the analyte during the process.

### 2.4. Sorption experiments

To conduct the sorption experiments, approximately 0.025 ± 0.002 g of dry polymer and 10 mL of a gentamicin solution with the concentration of 35 mg L ^−1^ were mixed at room temperature for 24 h. Following the achievement of equilibrium, the concentration of the antibiotic was determined by HPLC chromatography.

The amount of sorption at equilibrium, *Q*_e_ (mg g^−1^) was calculated using the following formula:1
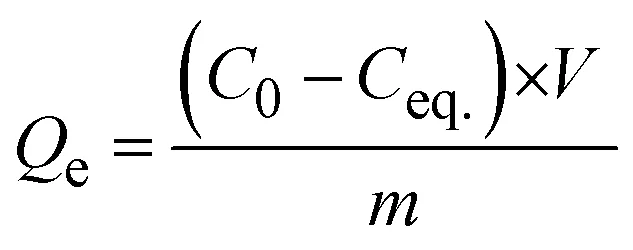
where *C*_0_ and *C*_eq._ (mg L^−1^) correspond to the initial and the final concentration of gentamicin, respectively. *V* (L) is referred as volume of the solution, while *m* (g) is referred as mass of the sorbents.^35,36^

#### 2.4.1. SPE columns

The sorption process was also carried out using SPE columns. For this purpose, approximately 0.1 g of sorbent (CS-MIP or CS-NIP) was weighed into a column with a lining of filter paper and cotton. These materials functioned as a filter within the column. The cartridges were subsequently activated with water, which also provided an additional step in the bed washing process. Thereafter, the dynamic sorption process was carried out by incubating the sorbent with a solution of gentamicin or a mixture of antibiotics for a period of 30 minutes.

### 2.5. Removal of gentamicin

Gentamicin desorption from the sorbent bed was performed in dynamic mode using water cooled to 4 °C as the elution solvent – in accordance with the PNIPAM smart mechanism. Following a 15-minute incubation period, the gentamicin concentration was quantified by HPLC. The regeneration efficiency, referred to as regeneration factor (*R*_f_), was calculated as follows:2
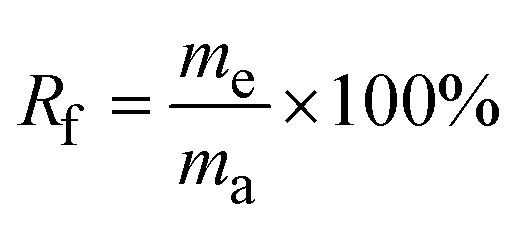
where *m*_e_ (mg) is mass of eluted gentamicin, and *m*_a_ (mg) is mass of absorbed gentamicin.

### 2.6. Sorption selectivity

The selectivity studies were conducted to evaluate the specific binding of gentamicin by CS-MIP. In order to ascertain the process, two other antibiotics (AMO and OFX) were selected and added to the solution of gentamicin. In the selective experiments, approximately 0.1 g of CS-MIP or CS-NIP was placed in the SPE column, after which 1 ml of antibiotic solution (26 mmol L^−1^ of each compound) was added. The sorption process was conducted for 30 minutes, and the concentration of gentamicin was determined by high-performance liquid chromatography (HPLC), while the concentrations of amoxicillin and ofloxacin were measured using ultraviolet-visible (UV-vis) spectroscopy. The selectivity was evaluated according to the distribution coefficient, imprinting factor, and selectivity factor.

The distribution coefficient (*K*) was calculated based on:3
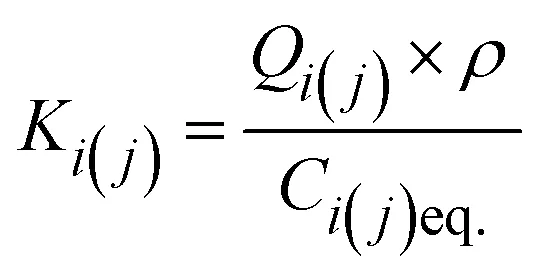
where *Q*_*i*(*j*)_ (µg g^−1^) is the sorption capacity (*i* – gentamicin, *j* – other antibiotics), *ρ* (g L^−1^) is the density of the solution, and *C*_*i*(*j*)eq._ (µg L^−1^) is the equilibrium concentration (*i* – gentamicin, *j* – other antibiotics).^35^

The imprinting factor (IF) was obtained from the following equation:4
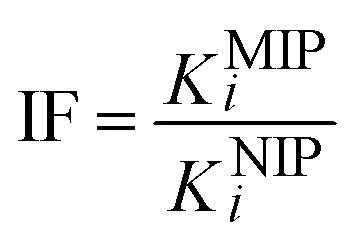
where *K*_*i*(*j*)_ is the distribution coefficient (*i* – gentamicin, *j* – other antibiotics) of an analyte on an imprinted polymer *K*^MIP^ (*K*^MIP^) and a non-imprinted polymer (*K*^NIP^).^34^

The selectivity factor (*S*) calculated by the following equation:5
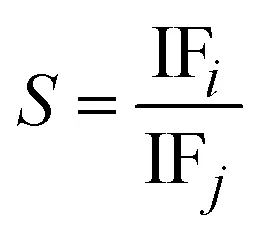
where IF_*i*_ and IF_*j*_ are imprinting factor of the gentamicin and other antibiotics, respectively.^35^

### 2.7. Effect of temperature on gentamicin removal

About 25 mg of NIP or MIP were weighed into test conical flasks. Subsequently, 10 mL of gentamicin solution, with a specified initial concentration about 35 mg L^−1^, was introduced into the flasks. The prepared samples were then placed in a laboratory incubator equipped with shaking (100 rpm), with the temperature set to either 277, 297, or 333 K. After 24 h, the concentration of gentamicin in the samples was analyzed with HPLC. To calculate the enthalpy change (Δ*H*°), entropy change (Δ*S*°), and change Gibbs free energy (Δ*G*°) were used from the van't Hoff (eqn (6)) and known relationship (eqn (7))^35^6
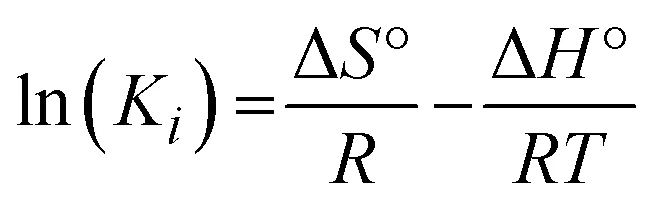

7 Δ*G*° = −*RT* ln(*K*_*i*_)

where *K*_*i*_ is the distribution coefficient (calc. from eqn (3)), *R* is the universal gas constant (8.314 J mol^−1^ K^−1^), and *T* is the temperature (K).

The values of Δ*H*° and Δ*S*° were determined from the slope and intercept, respectively, of the van't Hoff relationship between ln(*K*_d_) and 1/*T* using the experimental data obtained at 277, 297, and 333 K. These parameters were treated as constant over the investigated temperature range, whereas Δ*G*° was calculated separately for each temperature using eqn (7).

### 2.8. Real samples

The sorption of gentamicin from real tap water samples was also investigated under dynamic conditions. Approximately 0.1 g of sorbent (CS-MIP or CS-NIP) was packed into a column lined with filter paper and cotton. Prior to the sorption experiment, the cartridges were conditioned with water. Subsequently, the dynamic sorption process was conducted by passing a gentamicin solution through the column and incubating the sorbent for 30 minutes.

### 2.9. Material characterization

HPLC analysis was performed using a Vanquish™ Inverse Gradient LC System (ThermoFisher) equipped with a charged aerosol detector (CAD) and a Thermo Scientific Hypersil GOLD column (150 × 3 mm). The flow rate was set to 0.5 mL min^−1^, the column temperature was maintained at 25 °C, and the mobile phase consisted of 30% acetonitrile and 70% water. The injection volume was 30 µL. UV-vis absorbance spectra were recorded using a Specord 210 Plus spectrophotometer (Analytik Jena); amoxicillin and ofloxacin were quantified at 228 and 289 nm, respectively. The chemical structures of PVC, DHPVC, CS-MIP, and CS-NIP were characterized by Fourier-transform infrared spectroscopy (FT-IR) using a Jasco FT-IR-4700 spectrophotometer. Spectra were recorded over the range of 400–4000 cm^−1^ using 64 scans at a resolution of 4 cm^−1^. The morphology of DHPVC, CS-MIP, and CS-NIP was examined using a Talos L120C transmission electron microscope (TEM) after embedding the samples in epoxy resin and preparing ultrathin sections. Thermogravimetric analysis was performed using a TGA/DSC1 Mettler Toledo system by heating the samples from 25 to 800 °C at 10 °C min^−1^ under a nitrogen flow of 60 mL min^−1^. The specific surface area (*S*_BET_) and porosity-related parameters were determined from nitrogen sorption measurements at liquid nitrogen temperature using an ASAP 2020 Plus 2.00 analyzer. Prior to analysis, the samples were degassed at 50 °C for 10 h, and the nitrogen dose was adjusted to 3 cm^3^ g^−1^.

## 3. Results and discussion

### 3.1. Batch mode sorption process

In order to ascertain if the synthesised sorbents exhibit an affinity for gentamicin and possess the characteristics of temperature-sensitive smart polymers, a static sorption process was initially conducted at three different temperatures: 4, 24, and 60 °C. The results of the study are presented in Table 1. Temperatures of 4 and 60 °C were selected to provide a sufficiently large thermal stimulus and to evaluate whether temperature-induced changes in the NIPAM-containing polymer phase were sufficient to affect sorption and regeneration. The purpose of these experiments was therefore to assess the functional temperature response of the material rather than to determine the exact transition temperature of the copolymer layer. The maximum IF was observed at 24 °C, although the differences among the investigated temperatures were moderate. This trend is consistent with temperature-dependent changes in the PNIPAM-containing recognition phase. Additionally, the measurements performed at 24 °C also showed the lowest experimental variability.

**Table 1:** The sorption values carried out at different temperatures

	Temperature [°C]		
	4	24	60
CS-MIP (mg g^−1^)	3.59 ± 0.17	3.90 ± 0.07	3.58 ± 0.16
CS-NIP (mg g^−1^)	2.90 ± 0.23	2.80 ± 0.19	2.84 ± 0.21

The sorption capacity of the present CS-MIP reached 3.90 mg g^−1^ at 24 °C. This value is higher than that reported for a previously developed thermoresponsive MIP used in SPE columns,^30^ which showed a sorption capacity of 0.78 mg g^−1^ at ambient temperature, and also higher than that reported for a recently developed pH-responsive gentamicin MIP,^37^ for which a sorption capacity of 1.45 mg g^−1^ was obtained at pH 5.5. In contrast, a molecularly imprinted magnetic polymer developed for gentamicin removal from wastewater^38^ showed a substantially higher adsorption capacity of 60.21 mg g^−1^. However, a direct comparison of these values should be made with caution because the materials differ in architecture, experimental conditions, initial gentamicin concentration, and intended application.

We also considered the effect of pH on sorption and desorption properties. The p*K*_a_ for gentamicin falls within the range of 7.15–9.20.^39^ Consequently, at neutral pH, gentamicin carries a significant positive charge due to the protonation of the majority of its amino groups. Meanwhile, at a pH of approximately 4.50,^38^ acrylic acid dissociates primarily into the acrylate anion, and at higher pH values, it exists predominantly in the –COO^−^ form. Given this, conducting the process at neutral pH is advantageous, as it promotes strong interactions between the –COO^−^ and NH_3_^+^ groups. To achieve pH-triggered desorption, the process could be carried out in a strongly alkaline environment, under such conditions, the interactions between these groups weaken, facilitating the release of gentamicin molecules. We have previously reported on the influence of pH on the regeneration properties of polymers.^37^ The materials described in that earlier study did not contain PNIPAM in their structure, and their sorption properties were investigated at various pH levels. The presence of PNIPAM introduces an additional positive charge within the pH range of approximately 5.5–7. However, the molecular imprint formed during synthesis–also at a similar pH–ensures that the MIP-type material exhibits an affinity for gentamicin. This effect was confirmed in experiments using NIP material with the same composition but lacking the imprint.

The present CS-MIP was designed primarily for selective extraction and aqueous regeneration in an SPE-type system rather than for maximizing adsorption capacity.

### 3.2. Sorption-desorption cycles

In light of the prevailing focus on environmental protection and the production of MIPs in accordance with green molecular imprinting technology,^40,41^ this study investigates the potential for material reuse to reduce waste generation. The results are summarized in Fig. 1. Subsequent to each sorption cycle-comprising three stages for the first cycle and two stages for the second and third cycles-a desorption process, described as regeneration factor (*R*_f_), was conducted. The desorption process was carried out using the temperature-responsive behavior of the NIPAM-containing polymer phase. A temperature of 4 °C was selected to provide a strong thermal stimulus and promote hydration and expansion of the polymer network, thereby facilitating the release of gentamicin from the binding sites. In all sorption cycles, CS-MIP exhibited higher affinity for binding gentamicin. Following the initial cycle, it was observed that the first and second sorption processes allowed the majority of the binding sites on the polymer surface to be bound. In contrast, significantly smaller amounts of gentamicin molecules were bound following the third sorption stage. The total sorption value in the 1st cycle was 0.505 mg g^−1^ for CS-MIP and 0.257 mg g^−1^ for CS-NIP. Consequently, the sorption process in the second and third cycles was repeated twice. Furthermore, the sorption values of CS-MIP in the first and second cycles (0.423 and 0.261 mg g^−1^, respectively for CS-MIP and CS-NIP) are comparable. In the third cycle, however, the sorption value increased for both CS-MIP and CS-NIP but the difference in the sorption value of imprinted and non-imprinted polymers decreased significantly. This may be the result of irreversible conformational changes in the polymer layer chains caused by the smart mechanism. Perhaps, as a result of the subsequent conformational change, the imprinted cavities assumed a slightly different shape and lost some affinity for gentamicin. It is also possible that some degradation of the deposited layer occurred, making the surface less uniform and thus increasing physical sorption. For both sorbents, high desorption efficiencies were observed after the first and second cycles, reaching approximately 94–100%. After the third cycle, the desorption efficiency decreased for both CS-MIP (78%) and CS-NIP (72%). Despite the decrease observed, the fourth cycle was conducted. However, the expected results were not obtained, as it was impossible to sensibly determine the antibiotic concentration. Nevertheless, the materials can be reused up to three times for qualitative analysis and twice for quantitative analysis.

**Fig. 1:**
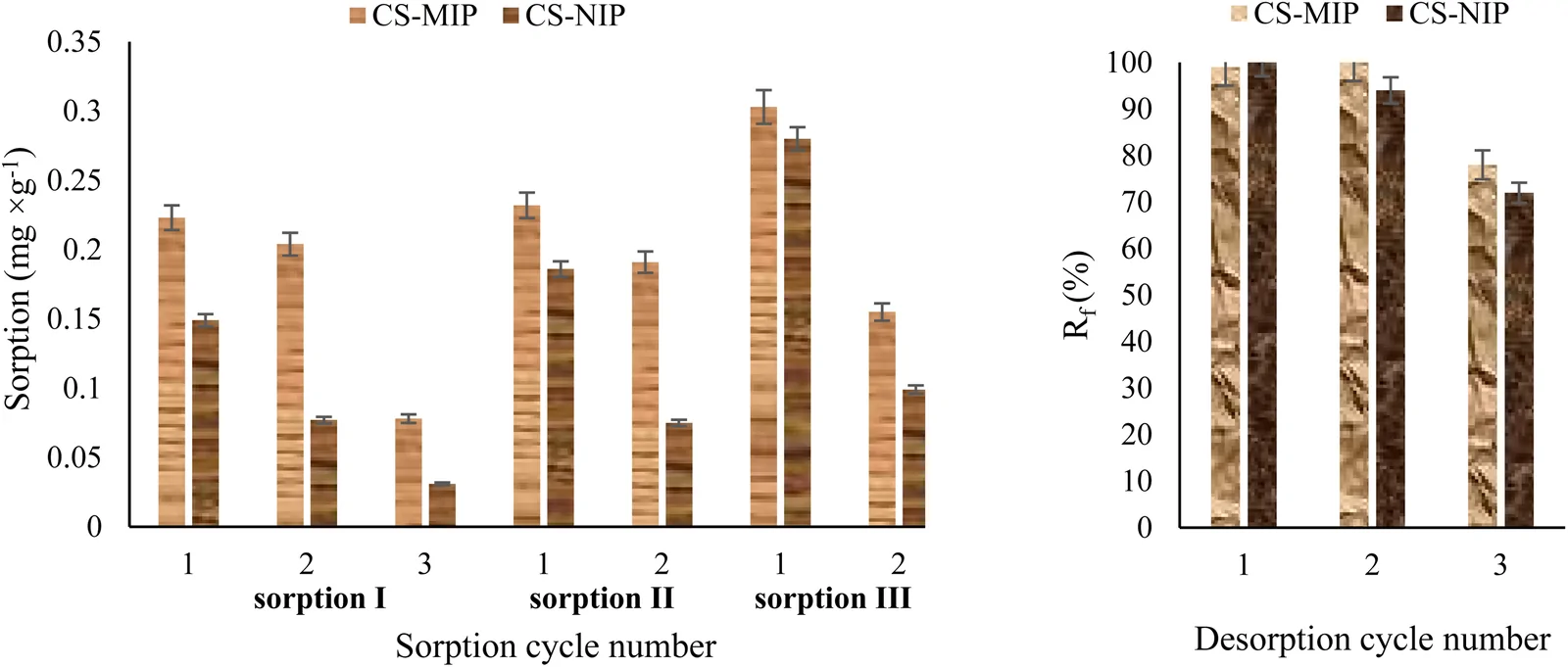
The results of the sorption (left) and desorption (right) cycles.

### 3.3. Specificity binding

It was checked if the CS-MIP matrix was verified to provide specific binding sites that interact with the gentamicin template. For these experiments-presented in Table 2, a series of antibiotics, in particular AMO and OFX, were employed. The imprinting factor for gentamicin (1.86) was higher than those obtained for amoxicillin (0.83) and ofloxacin (0.90), resulting in selectivity factors of 2.23 and 2.07, respectively. Since both *S* values were greater than 1, the results indicate preferential recognition of gentamicin by CS-MIP over the competing antibiotics and support the specificity of the imprinted binding sites. Furthermore, the *K* parameter approximately twofold increase for CS-MIP than CS-NIP, thereby supporting the presence of binding sites that are complementary to gentamicin.

**Table 2:** The results of the selectivity study results^a^

Parameter	Type of sorbent	GENT	AMO	OFX
*K*	CS-MIP	0.08	3.61	9.99
	CS-NIP	0.04	4.34	11.13
IF		1.86	0.83	0.90
*S*		—	2.23	2.07

a Not possible.

### 3.4. Effect of temperature on gentamicin removal

Conducting the sorption experiments at different temperatures allowed the determination of the thermodynamic parameters of the sorption process. The key thermodynamic parameters describing gentamicin sorption onto MIP and NIP were the enthalpy change (Δ*H*), the entropy change (Δ*S*), and the Gibbs free energy change (Δ*G*°). These parameters were calculated from the distribution coefficient (*K*_*i*_) using eqn (6) and (7). The Δ*H*° and Δ*S*° values were determined from the Van't Hoff relationship between ln(*K*_d_) and 1/*T* using the experimental data obtained at 277, 297, and 333 K. Therefore, the single Δ*H*° and Δ*S*° values reported for each material in Table 3 represent the investigated temperature range and were treated as constant within this range. In contrast, Δ*G*° was calculated separately for each temperature.

**Table 3:** Calculated thermodynamic parameters for the sorption of gentamicin^a^

Sample	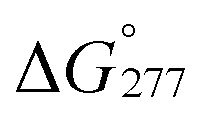 (kJ mol^−1^)	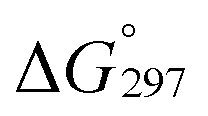 (kJ mol^−1^)	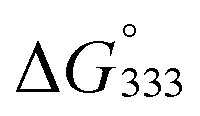 (kJ mol^−1^)	Δ*S*° (kJ mol^−1^ K^−1^)	Δ*H*° (kJ mol^−1^)
CS-NIP	−10.7	−11.4	−12.9	0.039	0.15
CS-MIP	−11.3	−12.4	−13.8	0.049	0.76

a Δ*H*° and Δ*S*° were determined from the van't Hoff relationship over the temperature range 277–333 K, whereas Δ*G*° was calculated separately for each investigated temperature.

The positive Δ*H*° values obtained for both MIP and NIP indicate that gentamicin sorption is an endothermic process. The relatively low Δ*H*° values suggest that only a small amount of energy was required for sorption. Consistently, the Δ*G*° values were negative for both materials at all investigated temperatures and became more negative with increasing temperature, indicating that sorption was spontaneous and thermodynamically more favorable at higher temperatures. Furthermore, the MIP exhibited more negative Δ*G*° values than the NIP, suggesting a stronger affinity of the imprinted polymer toward gentamicin and a more thermodynamically favorable sorption process. Additionally, the positive entropy changes (Δ*S*° = 0.039 and 0.049 kJ mol^−1^ K^−1^ for NIP and MIP, respectively) indicate an increase in the randomness at the solid-solution interface during sorption. The higher Δ*S*° value obtained for the MIP suggests a greater increase in disorder, which contributes to the enhanced spontaneity of the sorption process. It should be noted that, to the best of our knowledge, no thermodynamic study of gentamicin sorption onto molecularly imprinted polymers has been reported. Therefore, the obtained thermodynamic parameters were compared with those reported for other MIP-based sorption systems.

### 3.5. Application to real sample analysis

The core–shell molecularly imprinted polymers were applied to the determination of GENT in environmental water samples. The sorption process was performed three times and the results are presented in Fig. 2. In all three cycles, CS-MIP exhibited significantly higher gentamicin sorption than the CS-NIP, confirming the effectiveness of molecular imprinting. During the first sorption cycle, CS-MIP showed the highest uptake, reaching 0.43 mg g^−1^, whereas CS-NIP sorbed 0.17 mg g^−1^. In the second cycle, the sorption capacity decreased for both materials to around 0.18 mg g^−1^ for CS-MIP and 0.13 mg g^−1^ for CS-NIP. The third cycle showed the lowest sorption values, approximately 0.09 mg g^−1^ for CS-MIP and 0.05 mg g^−1^ for CS-NIP. The total sorption after three cycles was about 0.70 mg g^−1^ for CS-MIP and 0.35 mg g^−1^ for CS-NIP, which is half the sorption value of CS-MIP. Next, three desorption cycles were performed using water at a temperature of 4 °C, yielding an average regeneration factor of 95 ± 3% for CS-MIP and 98 ± 3% for CS-NIP. These data show that the material can be successfully used to determine gentamicin in real samples.

**Fig. 2:**
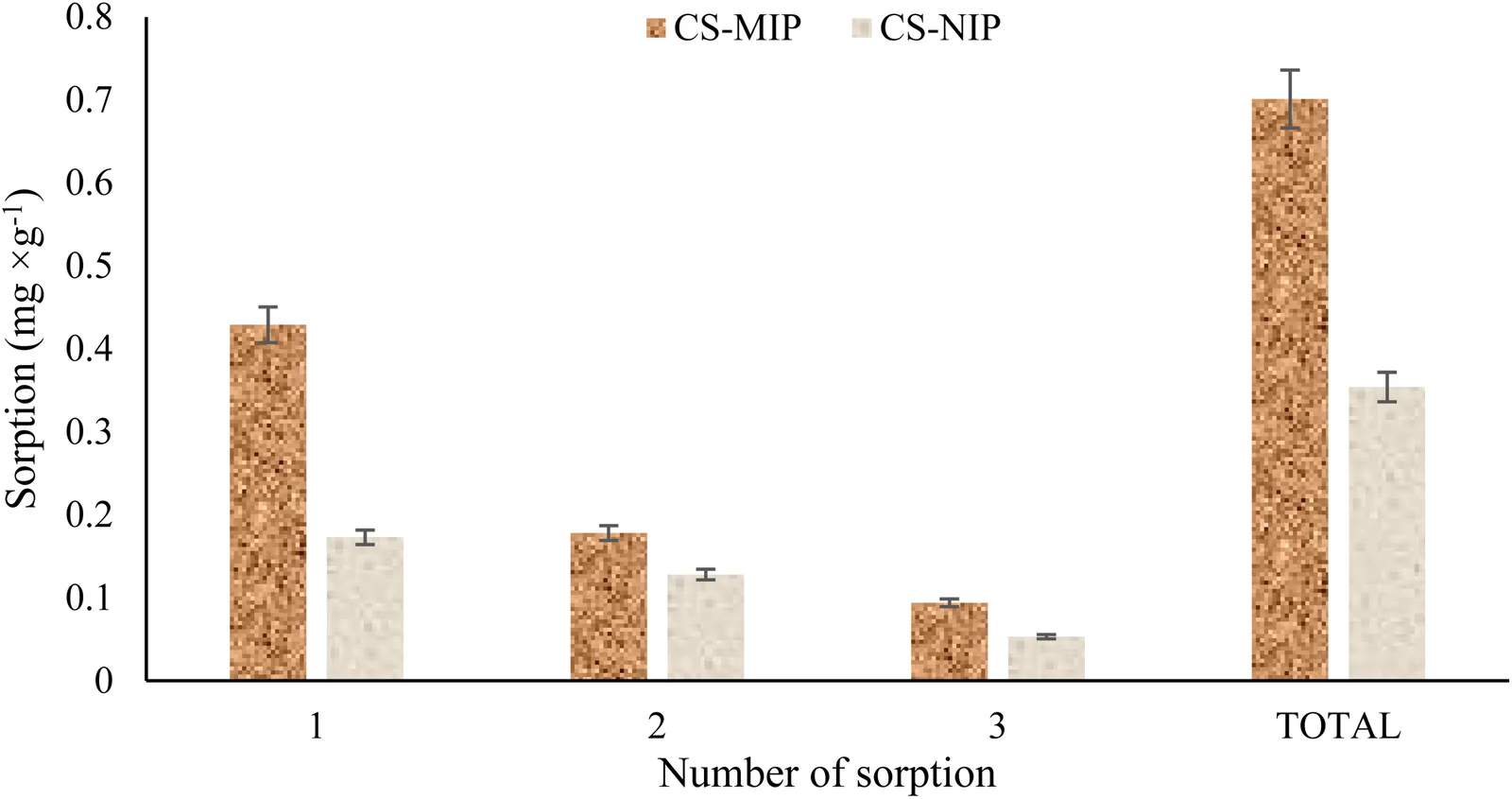
The results of gentamicin sorption from real samples.

### 3.6. FT-IR spectra

FT-IR spectra of PVC, DHPVC, CS-MIP and CS-NIP are compared in Fig. 3. The appearance of carbonyl- and amide-related bands in CS-MIP and CS-NIP confirms incorporation of the functional polymer phase into the modified DHPVC material. All spectra exhibit characteristic bands corresponding to C–H stretching vibrations at 2857 to 2983 cm^−1^, and CH_2_ deformation vibrations around 1413 cm^−1^. In the DHPVC spectra, a new band at 1640–1680 cm^−1^ was identified as a newly formed C=C bond. In the spectra of CS-MIP and CS-NIP, new absorption bands appeared as a result of the application of the polymer layer on the core. The prominent band at approximately 1680 cm^−1^ is attributed to the stretching vibration of carbonyl groups (C=O) from amide and carboxylic functionalities. The bands observed in the 1500–1650 cm^−1^ range are known to be associated with amide and C–N vibrations. The similar spectra of CS-MIP and CS-NIP confirm their comparable chemical composition, while the presence of carbonyl- and amide-related bands verifies the successful formation of the functional polymer shell on the DHPVC core.

**Fig. 3:**
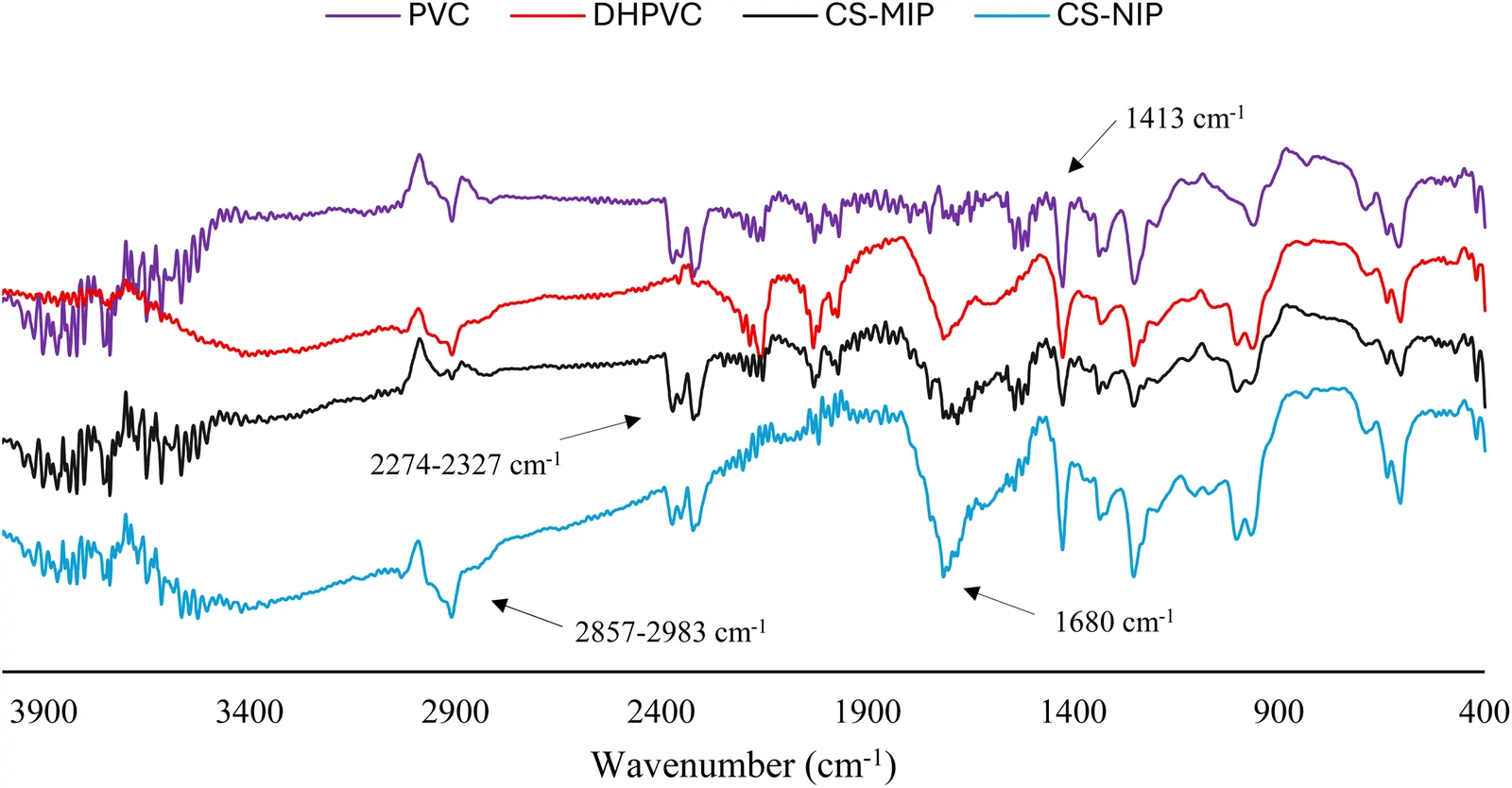
FT-IR spectra of polymers.

### 3.7. TEM characteristics

The resulting materials were imaged using a transmission electron microscope. The images are presented in Fig. 4. The CS-MIP and CS-NIP images do not reveal a sharply defined continuous layer surrounding the PVC core. Instead, regions of increased thickness are visible around the porous core structure. These features are consistent with modification of the near-surface region, although some penetration of the polymerization mixture into the porous substrate cannot be excluded. Therefore, the TEM data support the presence of a surface-associated polymer phase but do not allow the thickness or uniformity of a distinct shell to be determined. This limitation is important when interpreting the core–shell-type architecture of the materials.

**Fig. 4:**
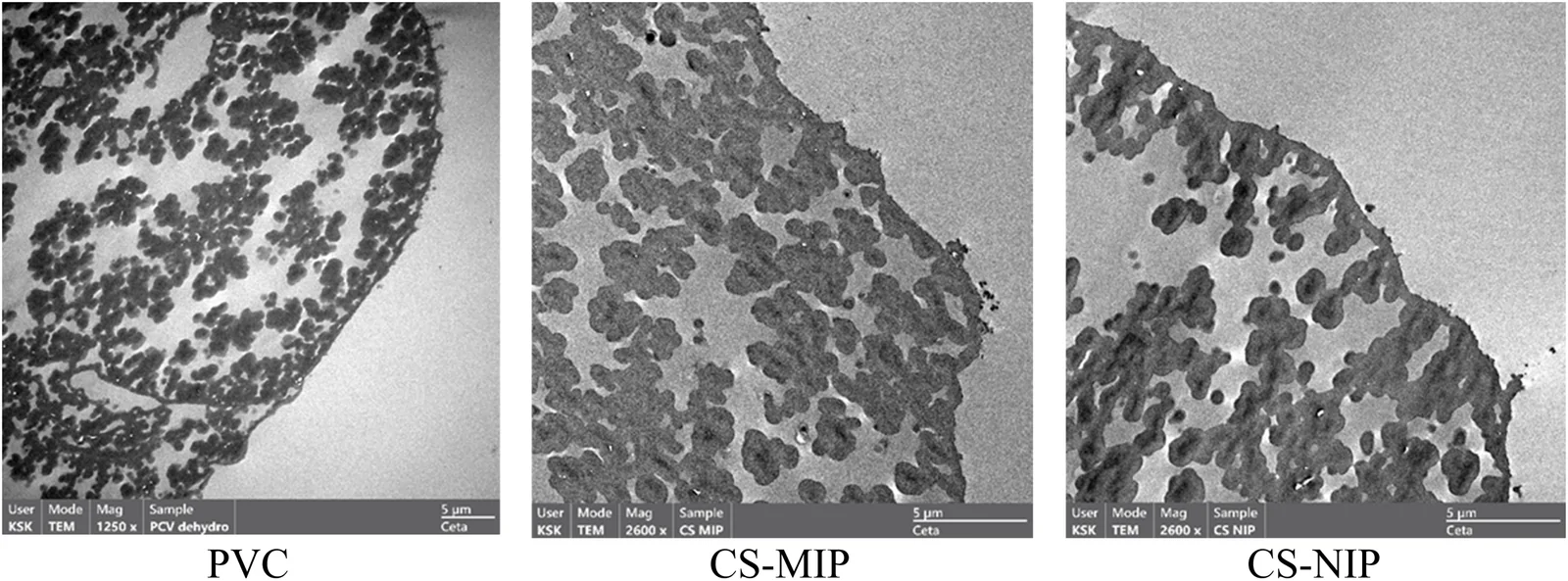
Transmission electron micrographs of sorbents.

### 3.8. Porosity

The specific surface area and sorption parameters were determined to assess the influence of the polymer coating and molecular imprinting process on the properties of the materials. As illustrated in Table 4, the BET analysis showed that both CS-MIP (2.42 m^2^ g^−1^) and CS-NIP (2.45 m^2^ g^−1^) exhibited higher specific surface areas in comparison to the PVC core (1.74 m^2^ g^−1^). A similar tendency was noted in the single-point surface area and sorption capacity. The *Q*_m_ values for CS-MIP and CS-NIP are comparable, which may be due to the fact that the applied polymer layer is very thin. However, the deposition of the polymer layer onto the PVC core resulted in an increase in the surface area of the material. Despite the presence of similar parameters, CS-MIP still exhibits enhanced sorption selectivity due to the specificity of the recognition sites. It was not possible to determine the average pore size of the polymers due to the very small surface area.

**Table 4:** The porosity study results

Type of polymer	BET surface area (m^2^ g^−1^)	Single point surface area (m^2^ g^−1^)	*Q*_m_ (cm^3^ g^−1^)
CS-MIP	2.417	2.281	0.555
CS-NIP	2.445	2.301	0.562
PVC	1.742	1.696	0.400

### 3.9. TGA analysis

In order to compare the obtained materials and to check the effect of modification, a thermogravimetric analysis was performed. The sample mass was normalized to the initial mass at the beginning of the measurement (approximately 29 °C), which was defined as 100%. The corresponding derivative thermogravimetric (DTG) curves were used to identify the main decomposition regions (Fig. 5). For all three materials, two main stages of mass loss were observed. While the thermograms of CS-MIP and CS-NIP overlap, slight differences can be observed for PVC. At low temperatures, only a small mass loss occurred, which can be attributed mainly to the release of physically sorbed moisture. The absence of a pronounced low-temperature mass-loss step indicates that the materials contained only a limited amount of moisture after drying. The first major mass-loss region occurred approximately within the 200–350 °C range, with the highest degradation rate observed near 276 °C. For the PVC core, this stage is mainly associated with dehydrochlorination of the polymer backbone. A second major degradation region was observed at higher temperatures, with a DTG maximum around 431 °C. This stage can be associated with further degradation of the PVC structure and decomposition of the remaining polymeric backbone. At higher temperatures, the residual mass approached approximately 18–20%, which under nitrogen can be attributed mainly to thermally stable carbonaceous char. The TGA and DTG profiles of CS-MIP and CS-NIP were very similar, indicating that molecular imprinting did not substantially change the overall thermal degradation pathway. Small differences between the modified materials and the PVC core indicate the contribution of the deposited functional polymer phase. The apparent upward transitions in the DTG curves do not represent an increase in sample mass but reflect changes in the rate of mass loss between consecutive decomposition stages.

**Fig. 5:**
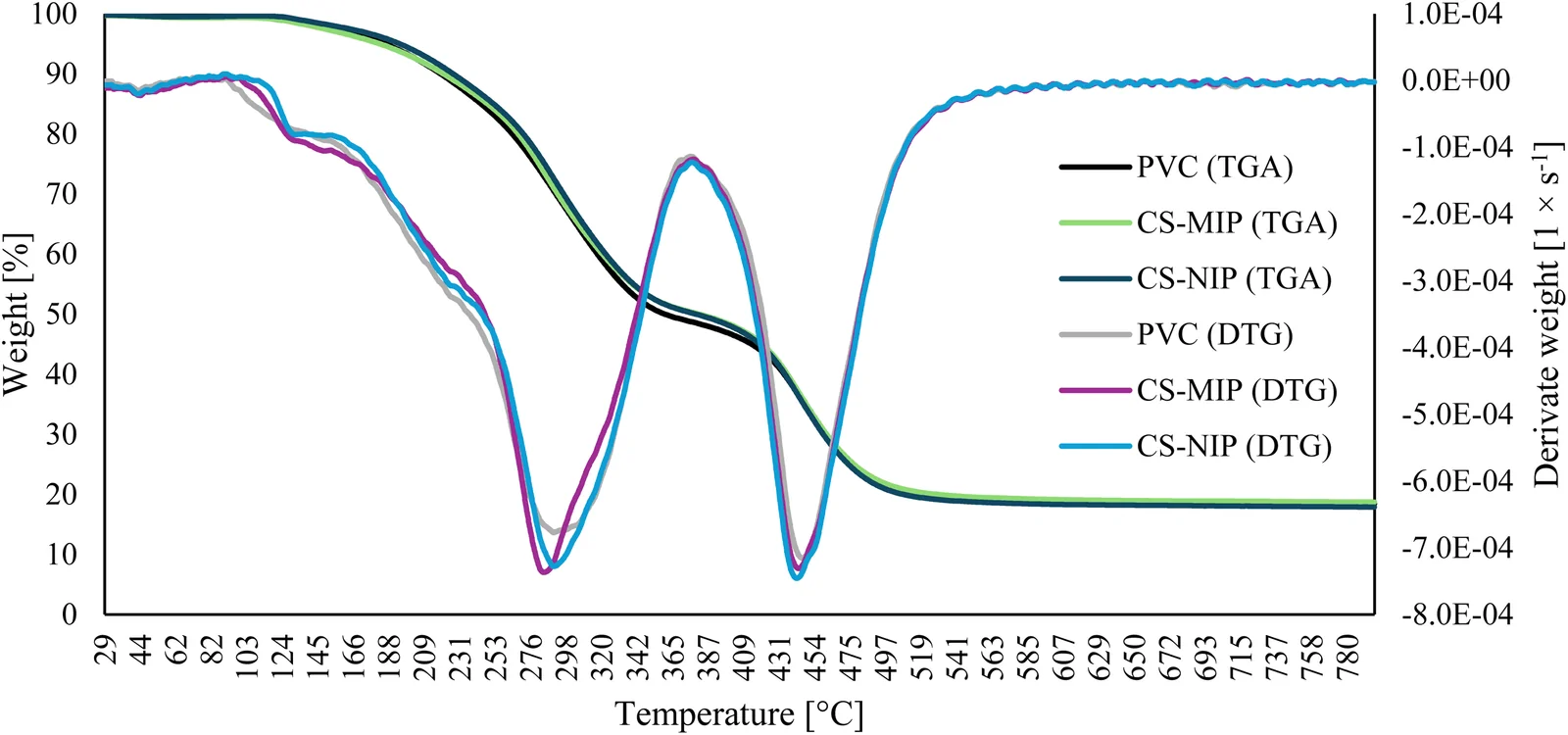
TGA and DTG curves of PVC, CS-MIP, and CS-NIP.

## 4. Conclusions

A thermoresponsive, surface-localized molecularly imprinted sorbent was developed for gentamicin recognition and temperature-controlled aqueous regeneration. The CS-MIP exhibited a higher sorption capacity than CS-NIP, reaching 3.90 mg g^−1^ with an imprinting factor of 1.40 under batch conditions. Preferential recognition of gentamicin was also observed in the presence of competing antibiotics. Cooling to 4 °C enabled efficient desorption using water, with regeneration efficiencies of 94–100% during the first two cycle. The material was successfully applied to gentamicin extraction from tap water samples, demonstrating its potential practical sample preparation in aqueous matrices. Although the sorption and regeneration performance decreased upon repeated reuse, the material remained suitable for multiple analytical cycles. Overall, the combination of surface-localized recognition sites, thermoresponsive behavior, and water-based regeneration makes CS-MIP a promising reusable sorbent for SPE-based extraction of gentamicin with reduced use of organic solvents.

## Conflicts of interest

There are no conflicts to declare.

## Data Availability

All data necessary to understand and support the findings of this study are included in the article. The underlying raw experimental data will be deposited in the RepOD repository and made publicly available upon publication of the article.
